# C-953-05. From Foundation to Excellence: Constructing a Strong and Productive Burn Surgery Advanced Practice Provider Team

**DOI:** 10.1093/jbcr/irag033.129

**Published:** 2026-04-06

**Authors:** Christopher T Borchers, Michael J Feldman, Lesley B Coots

**Affiliations:** Virginia Commonwealth University, Richmond, VA; Virginia Commonwealth University, Richmond, VA; Virginia Commonwealth University, Richmond, VA

## Abstract

**Introduction:**

Burn centers face a growing need to provide optimal patient care with proficient providers. In 2020, we began to assemble a Burn Surgery Advanced Practice Provider Team (APPT), to provide 24/7/365 coverage. Building and sustaining this team required a strategic approach integrating recruitment, training, collaboration, and continuous development.

**Methods:**

A Burn Surgery APPT was created to provide 24-hour coverage of in-patients, out-patients, consults, and operative cases. APPs were recruited with a focus on highly skilled and motivated professionals. Onboarding and training were essential to equip Burn Surgery APPs with skills and knowledge in burn care, surgical technique, and patient management. This process included a 12-week orientation to the Burn Surgery Service.

**Results:**

In 2020, we employed four APPs (4.0 FTE) who only covered dayshift. In 2024, we employ, 17 APPs (12.1 FTE), with a turnover of only one APP. We provide 7 day, 24-hour coverage of inpatients, burn consults, operative cases and transfer center calls; the outpatient clinic is staffed by two full-time APPs who each see approximately 16 patients daily, five days per week. APPs function in multiple roles, practicing at the top of their scope, increasing role satisfaction and professional growth.

In 2023, APPs were surveyed. All APPs (630) and Plastics/Burn Surgery (PBS) APPs (11) (Response Rate, n = 5, 45%) using Press Ganey indicators of Engagement (a measure of provider workplace satisfaction) and Alignment (a measure of provider value). The engagement indicator for the Hospital was 3.78, and the PBS was 3.77. The alignment indicator for the Hospital was 3.37, and the PBS was 3.78.

Increased financial gain was observed. Burn Relative Value Units (RVUs) has increased annually with Burn APP growth. Fiscal year (FY) 2022 saw five Burn APPs produce 5191.75. While FY 2024 saw 12.4 Burn APPs produce 8086 RVUs.

From June 2022 – May 2023, the Burn Surgery 30 day and 7 day re-admission rates were, 20.22% and 8.08%, respectively. Over the course of the following year, May 2023- June 2024, these rates fell to 5.90% and 2.17%, respectively.

**Conclusions:**

Developing and maintaining a high-functioning Burn Surgery APPT is vital to a successful Burn Program. Unique staffing models allow Burn Surgery APPTs to meet both patient and their own team members by offering work/life balance. A proficient Burn Surgery APPT engaged and aligned with the institution can improve role satisfaction and decrease APP turnover. Ensuring APPs practice to the top of their scope contributes to role satisfaction, team efficiency, and productivity.

**Applicability of Research to Practice:**

Building a robust, successful Burn Surgery APPT takes time and buy-in from hospital leadership to support the growth. Burn Surgery APPTs can contribute to productivity in innovative ways to offload Burn Surgeons, such as laser therapy, staffing clinics and assisting in all aspects of inpatient burn care.

**Funding for the study:**

N/A.

